# Investigating the Accuracy and Consistency of ChatGPT in the Management of Achilles Tendon Ruptures

**DOI:** 10.7759/cureus.78433

**Published:** 2025-02-03

**Authors:** Christopha J Knee, Ryan J Campbell, Brahman S Sivakumar, Andrew Wines, Michael J Symes

**Affiliations:** 1 Department of Orthopaedics and Trauma Surgery, Royal North Shore Hospital, Sydney, AUS; 2 Department of Hand and Peripheral Nerve Surgery, Royal North Shore Hospital, Sydney, AUS

**Keywords:** achilles tendon, artificial intelligence, chatgpt, rupture, tendoachilles

## Abstract

Background

The emergence of generative artificial intelligence, such as ChatGPT (OpenAI, San Francisco, CA, USA), offers significant potential for improving the delivery of patient information and aiding in clinical decision-making. The aim of this study was to investigate the accuracy and consistency of ChatGPT in providing patient information and answering orthopaedic clinical questions regarding Achilles tendon ruptures.

Methods

Eight questions regarding Achilles tendon rupture management were presented to ChatGPT twice, resulting in 16 responses. References were requested for all responses. Each response was evaluated for accuracy and consistency, utilising a grading scale ranging from I (comprehensive) to IV (completely incorrect). Final grading was determined through consensus discussions among two orthopaedic registrars and two senior orthopaedic surgeons. Descriptive statistics were performed.

Results

All of the responses produced by ChatGPT were graded as containing both correct and incorrect information (grade III). Consistency was observed in six out of eight (75%) questions when comparing the two responses for each question. ChatGPT provided 47 references, with 16 out of 47 (34%) correct, 19 out of 47 (40%) incorrect, and 12 out of 47 (26%) fabricated.

Conclusion

ChatGPT lacks accuracy and consistency in providing information on the management of Achilles tendon ruptures. All patient information and orthopaedic clinical decision-making recommendations contained inaccurate or fabricated information.

## Introduction

Achilles tendon ruptures are common, primarily occurring in the third to fifth decades of life, due to participation in high-demand sports or recreational activities [1]. These injuries are over three times more common in males, with an incidence rate ranging from three to 55 per 100,000 person-years, and can be debilitating to locomotion and daily function [1-4]. In sporting populations, up to 25% of elite athletes do not return to sport following an Achilles tendon rupture [5].

These injuries can be managed operatively (either open or percutaneous approaches) or non-operatively, via functional accelerated rehabilitation. Although traditionally treated via surgical intervention in younger patients, evidence pointing to decreased differences between the two modalities (and increased complications with operative management) have resulted in a paradigm shift toward non-operative measures [6-10].

ChatGPT, developed by OpenAI (San Francisco, CA, USA), is a generative artificial intelligence chatbot. It belongs to the category of large language models (LLMs), trained on extensive datasets to mimic human-like language and communication [11]. ChatGPT has demonstrated impressive medical knowledge, achieving scores at or near the passing threshold for the United States Medical Licensing Examination (USMLE) [12,13]. It exhibits medical reasoning consistent with experts, provides clinically relevant information for common knee and shoulder orthopaedic conditions, and has demonstrated utility in medical education, clinical workflow, and patient education [13-16]. Furthermore, ChatGPT has outperformed doctors' responses in online patient queries regarding quality and empathy [17].

While ChatGPT enjoys worldwide popularity and shows promise in medical contexts, it has been criticised for producing inaccurate content, often called "'hallucinations", and for making frequent errors [18-20]. Currently, there is limited research on how ChatGPT provides information on orthopaedic conditions. This study aims to assess ChatGPT's ability to provide accurate and consistent information on Achilles tendon ruptures for patients and to aid in orthopaedic clinical decision-making.

## Materials and methods

Study design

ChatGPT 3.5 was presented with a series of eight questions (Table 1), covering varying aspects of Achilles tendon rupture management.

**Table 1:** ChatGPT input questions on the management of Achilles tendon ruptures

Patient-orientated questions
1. In 200 words, what are the treatment options for a complete rupture of the Achilles tendon? Please include references from three high-quality journal articles, ensuring that these references are cited both within the text and in a separate reference list.
2. In 200 words, when is surgery indicated for rupture of the Achilles tendon? Please include references from three high-quality journal articles, ensuring that these references are cited both within the text and in a separate reference list.
3. In 200 words, is rehabilitation important after surgical and non-surgical treatment of a complete rupture of the Achilles tendon? Please include references from three high-quality journal articles, ensuring that these references are cited both within the text and in a separate reference list.
4. In 200 words, compare the recovery time and re-rupture rate between non-surgical and surgical treatment of a complete rupture of the Achilles tendon. Please include references from three high-quality journal articles, ensuring that these references are cited both within the text and in a separate reference list.
Orthopaedic clinical questions
5. I'm an orthopaedic registrar seeking advice for a 33-year-old male who has a complete rupture of his Achilles tendon. He works in an office and maintains a low level of physical activity. Should I recommend surgical or non-surgical treatment? Please provide a 150-word recommendation, supported by high-quality references. Ensure the inclusion of both in-text citations and a separate reference list.
6. I'm an orthopaedic registrar seeking advice for a 25-year-old male elite basketball player who has a complete rupture of his Achilles tendon. He needs to return to sport as quickly as possible. Should I recommend surgical or non-surgical treatment? Please provide a 150-word recommendation, supported by high-quality references. Ensure the inclusion of both in-text citations and a separate reference list.
7. I'm an orthopaedic registrar seeking advice for a 33-year-old female who has a complete rupture of her Achilles tendon. She presents 6 weeks after the injury with a clinically palpable defect and a 3cm gap observed on ultrasound during full ankle plantarflexion. Should I recommend surgical or non-surgical treatment? Please provide a 150-word recommendation, supported by high-quality references. Ensure the inclusion of both in-text citations and a separate reference list.
8. I'm an orthopaedic registrar seeking advice for a 67-year-old male who has an open and complete rupture of his Achilles tendon. He leads a mostly sedentary lifestyle but does spend some time in the garden. Should I recommend surgical or non-surgical treatment? Please provide a 150-word recommendation, supported by high-quality references. Ensure the inclusion of both in-text citations and a separate reference list.

Questions 1-4 aimed to replicate patient queries commonly searched on Google. To anticipate patient questions, Google Trends was used to search for "ruptured Achilles tendon" and "torn Achilles tendon", identifying related worldwide health queries in web searches from 2004 to the present. Google Trends highlights the most popular Google search engine queries related to a particular topic. Additionally, it provides the capability to track the progression of search terms following an initial query, a feature known as "related queries". The top Google search terms included "treatment", "surgery", "recovery", and "rupture" of the Achilles tendon, which were used in formulating patient questions.

Questions 5-8 were designed to simulate clinical inquiries that an orthopaedic clinician might present to ChatGPT to aid decision-making. The management domains explored encompassed surgical versus non-surgical treatment, the management of elite athletes, delayed (neglected) Achilles tendon ruptures, and open Achilles tendon injuries. For all questions, high-quality references were requested to ascertain the basis and accuracy of the information or recommendation provided.

Each question was entered twice on February 6, 2024, to evaluate response accuracy and consistency. To ensure previous interactions did not influence subsequent responses, each question was submitted as a distinct "New Chat" in a new session. All responses were recorded as screenshots and are included in the Appendices.

Institutional ethics approval was deemed unnecessary for this study, as it did not involve human or animal subjects and because the information that ChatGPT provides is publicly available.

Assessment of responses

Responses were independently evaluated for both accuracy and consistency by two orthopaedic registrars (CK and RC) and two senior orthopaedic surgeons (MS and BS). Each response was assessed using a modified accuracy grading scale previously applied in the literature: (I) comprehensive, (II) correct but inadequate, (III) mixed with correct and incorrect information, and (IV) completely incorrect [21]. Consistency was defined as similar accuracy grading, response content, and recommendations across both responses for each of the eight questions, assigning a dichotomous grade of "yes" or "no". Discrepancies in grading were discussed until a consensus was reached.

The accuracy of the references provided in the responses was verified by conducting searches across MEDLINE, Embase, and Scopus. References were deemed incorrect if they contained inaccurate details such as the title, authors, date of publication, or journal information or were considered fabricated if the reference could not be found.

Statistical analysis

Descriptive statistics, including frequencies, counts, percentages, and the standard error of the mean (SEM) for relevant variables, were calculated using Microsoft Excel (Version 16.77, Microsoft Corporation, Redmond, WA, USA).

## Results

ChatGPT response characteristics

**Figure 1:**
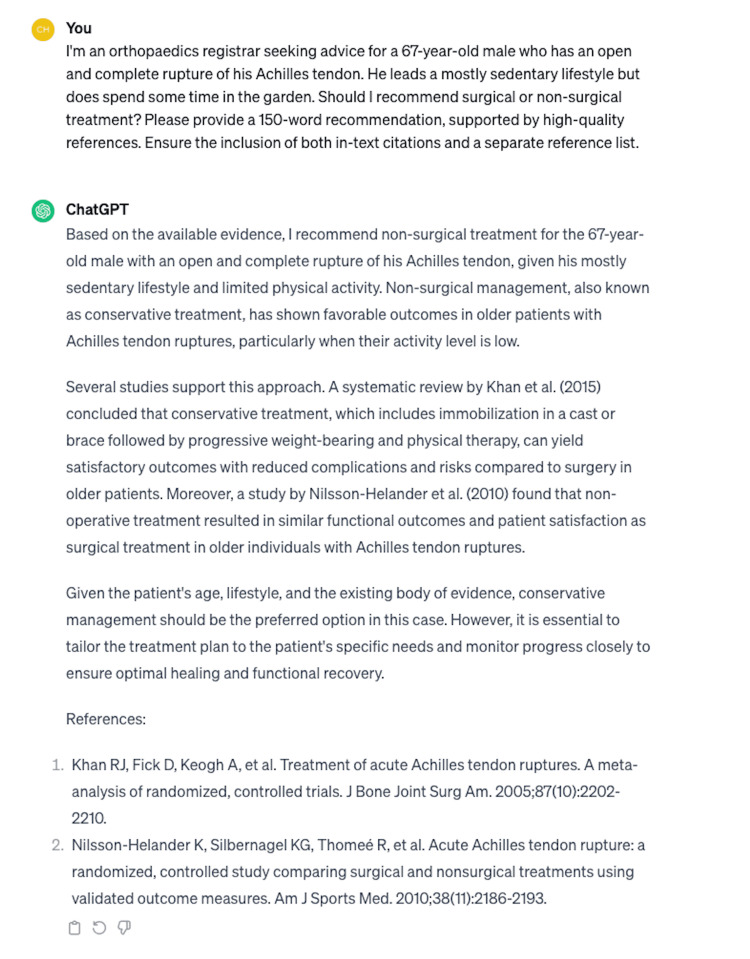
Example of ChatGPT response (question 8, response 2)

Eight questions were presented to ChatGPT on two separate occasions, producing a total of 16 unique responses. The mean length of the patient-oriented responses (from questions 1 to 4) was 217 ± 10 words (SEM; range: 182-250). For clinical decision-making responses (from questions 5 to 8), the mean length was 165 ± 6 words (SEM; range: 135-182). An example of a ChatGPT response is provided in Figure 1.

Information accuracy and consistency

The accuracy of all 16 responses was graded as III, indicating a mix of correct and incorrect information. Consistency was observed in six out of eight questions (75%) when comparing the two responses provided for each question (Table 2).

**Table 2:** Evaluation of accuracy and consistency of ChatGPT responses Grades I-IV: I, comprehensive; II, correct but inadequate; III, mixed with correct and incorrect information; and IV, completely incorrect Q: question; R1: first response; R2: second response; n: number; nt: total number; Y: yes; N: no

Question	R1 grade (I-IV)	R1 reference accuracy, n/nt	R2 grade (I-IV)	R2 reference accuracy, n/nt	Consistency (Y/N)
Q.1	III	0/3	III	0/3	Y
Q.2	III	0/3	III	0/3	Y
Q.3	III	0/3	III	1/3	Y
Q.4	III	4/4	III	1/3	Y
Q.5	III	1/3	III	2/4	N
Q.6	III	2/2	III	0/3	Y
Q.7	III	2/2	III	0/2	Y
Q.8	III	1/4	III	2/2	N

Reference analysis

Across the 16 responses, ChatGPT cited 47 references: 16 (34%) were correct, 19 (40%) were incorrect, and 12 (26%) appeared to be fabricated.

## Discussion

This study highlights numerous limitations of ChatGPT in providing accurate and consistent information to patients and assisting in orthopaedic clinical decision-making regarding Achilles tendon ruptures. All 16 responses contained a mix of correct and incorrect information (grade III). None of the responses were answered comprehensively.

The results of our study bear similarities to the findings of Cuthbert and Simpson [22], who reported ChatGPT's inability to exert higher-order judgment and logical thinking when answering orthopaedic surgery and trauma-related examination questions. ChatGPT was asked a total of 134 orthopaedic surgery and trauma-related questions and answered only 48 questions correctly (35.8%). Notably, it failed to answer any of the 15 questions related to trauma correctly.

ChatGPT frequently made referencing errors, with 31 out of 47 (66%) of the references provided to support its advice and recommendations containing incorrect or fabricated details. It often misquoted studies and inaccurately claimed the superiority of interventions not supported by its provided references. The fabrication of references by ChatGPT has consistently been observed by other authors [18,19]. OpenAI cautions users against ChatGPT's tendencies to hallucinate, fabricate facts, and generate incorrect information [23]. This is concerning as ChatGPT becomes increasingly difficult to distinguish from medical professionals [17].

Similar to other studies, ChatGPT was found to be user-friendly, provided quick responses, and was interactive when answering patient-orientated questions [18,19,21]. It promoted collaborative decision-making with patients and highlighted crucial decision-making criteria for managing Achilles ruptures, including age, overall health, functional demands, and patient objectives. ChatGPT effectively distinguished between surgical and non-surgical management options and emphasised the importance of rehabilitation for optimal outcomes, irrespective of the chosen management.

However, all responses to patient-orientated questions contained incorrect information, often diverging significantly from the true conclusions of cited sources, resulting in contradictions within and between responses. For example, in the first response to question 2, ChatGPT cited a study by Khan et al. [24] indicating superior functional outcomes and reduced re-rupture rates with surgical management of Achilles tendon ruptures. Yet, in a subsequent reference to the same paper in question 4 (response 1), ChatGPT asserted that re-rupture rates were similar between surgical and non-surgical treatments. Furthermore, in the same response, it was incorrectly suggested that non-surgical treatment led to higher complication rates and a faster return to work and daily activities, which contradicts the true findings of the cited papers [25,26].

In a study conducted by Rao et al. [27], ChatGPT demonstrated a 71% accuracy rate in clinical decision-making across 36 standardised medical vignettes. In contrast, this study observed less favourable results. ChatGPT exhibited both inaccuracies and inconsistencies in assisting with orthopaedic clinical decisions regarding Achilles tendon ruptures. Notably, in both questions 5 and 8, ChatGPT initially recommended surgery but later opposed it in subsequent responses. It also failed to recognise that an open Achilles rupture is an absolute indication for surgical intervention (Figure 1).

Given that the current evidence base demonstrates comparable functional outcomes between surgical and non-surgical management of Achilles tendon ruptures, it was expected that ChatGPT would present a balanced discussion and recommend non-surgical management for most patients, with the exception of those with open Achilles ruptures, elite athletes, or those with delayed presentations (neglected Achilles tendon ruptures) [6-10]. However, ChatGPT's recommendations were mixed, inaccurate, and often not based on the cited references. For example, when recommending surgery, ChatGPT frequently cited studies that reported no significant clinical differences between surgical and non-surgical management [28].

A strength of this study is the use of Google Trends to identify commonly searched terms, ensuring that patient-focused questions reflect real-world inquiries. Additionally, the inclusion of four independent reviewers (two junior and two senior orthopaedic surgeons) reduces bias and subjectivity in the assessment process.

However, this study had notable limitations. ChatGPT 3.5, the most accessible and free version of ChatGPT in Australia, was used; other versions may yield different results. Additionally, the way references were requested, both in text and in a reference list, could have affected the quality of ChatGPT's responses. Limiting the number of references requested in questions 1-4 may have also influenced the nature of the references provided. The replicability of our findings depends on the phrasing of questions, which may not represent the health literacy of all patients.

## Conclusions

ChatGPT lacks accuracy and consistency in providing information on the management of Achilles tendon ruptures. All patient information and orthopaedic clinical decision-making recommendations contained inaccurate or fabricated information. These findings suggest that caution should be used by patients and clinicians when using ChatGPT.

## Figures and Tables

**Figure 2:**
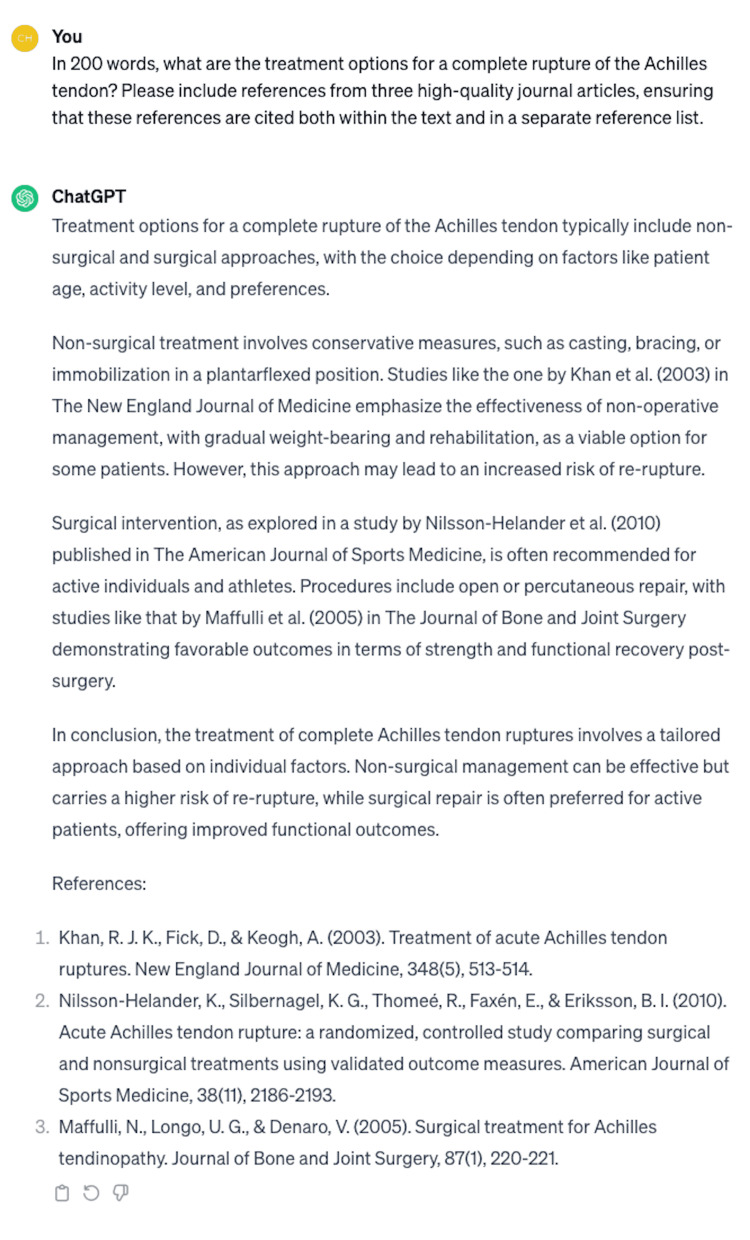
ChatGPT question 1, response 1

**Figure 3:**
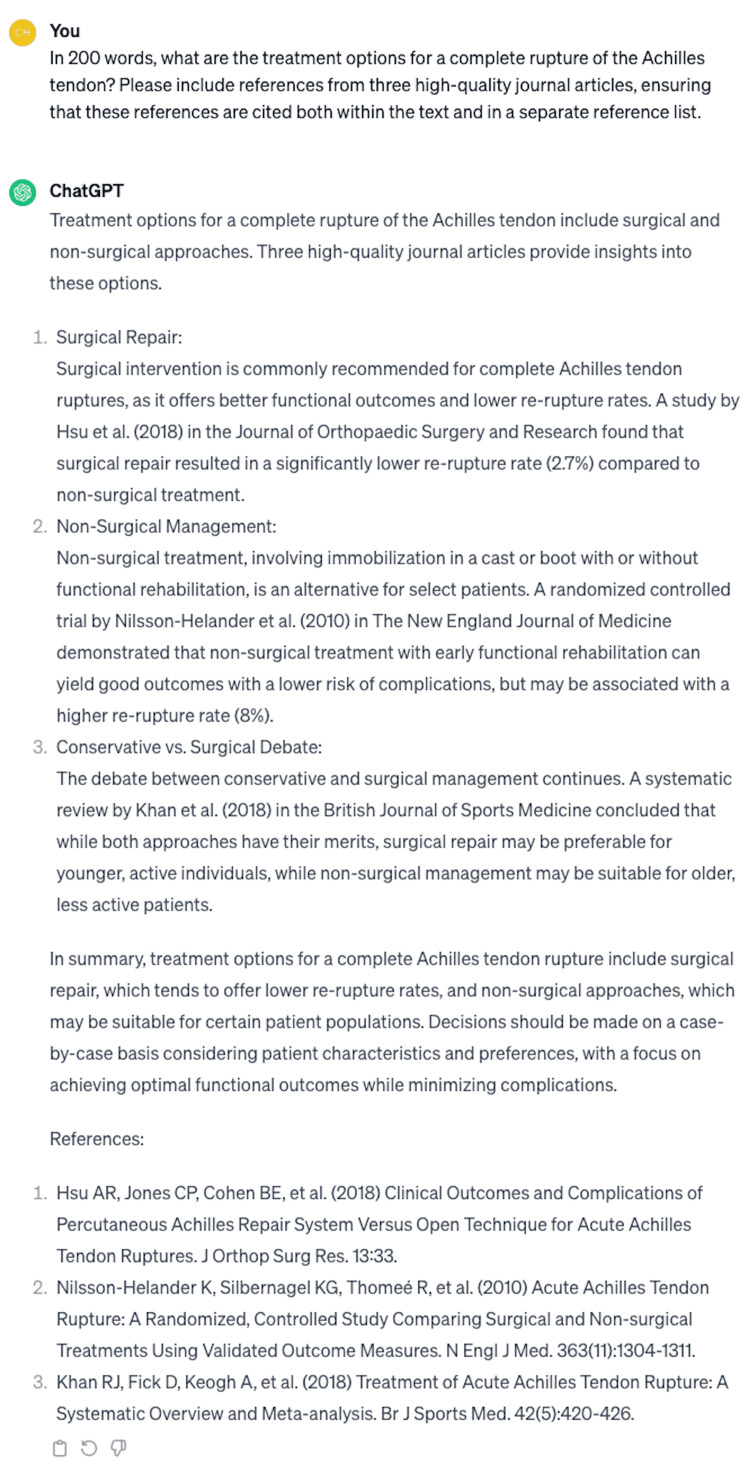
ChatGPT question 1, response 2

**Figure 4:**
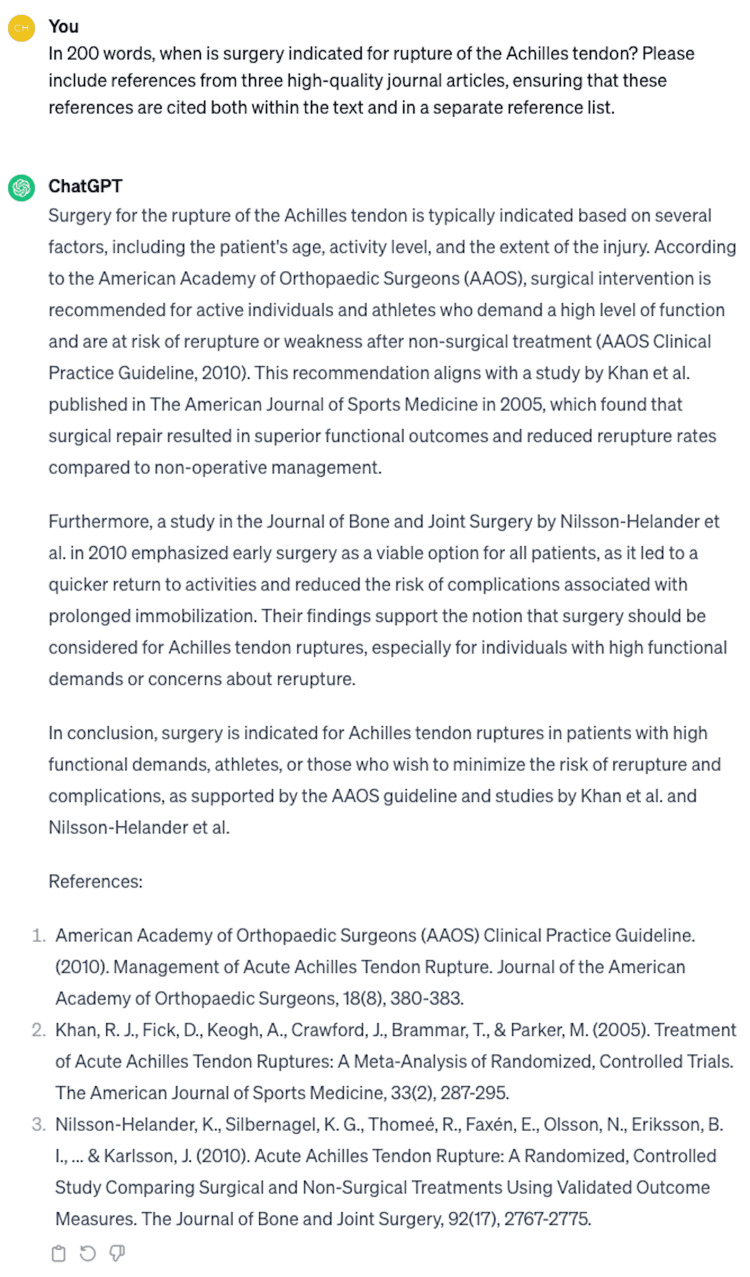
ChatGPT question 2, response 1

**Figure 5:**
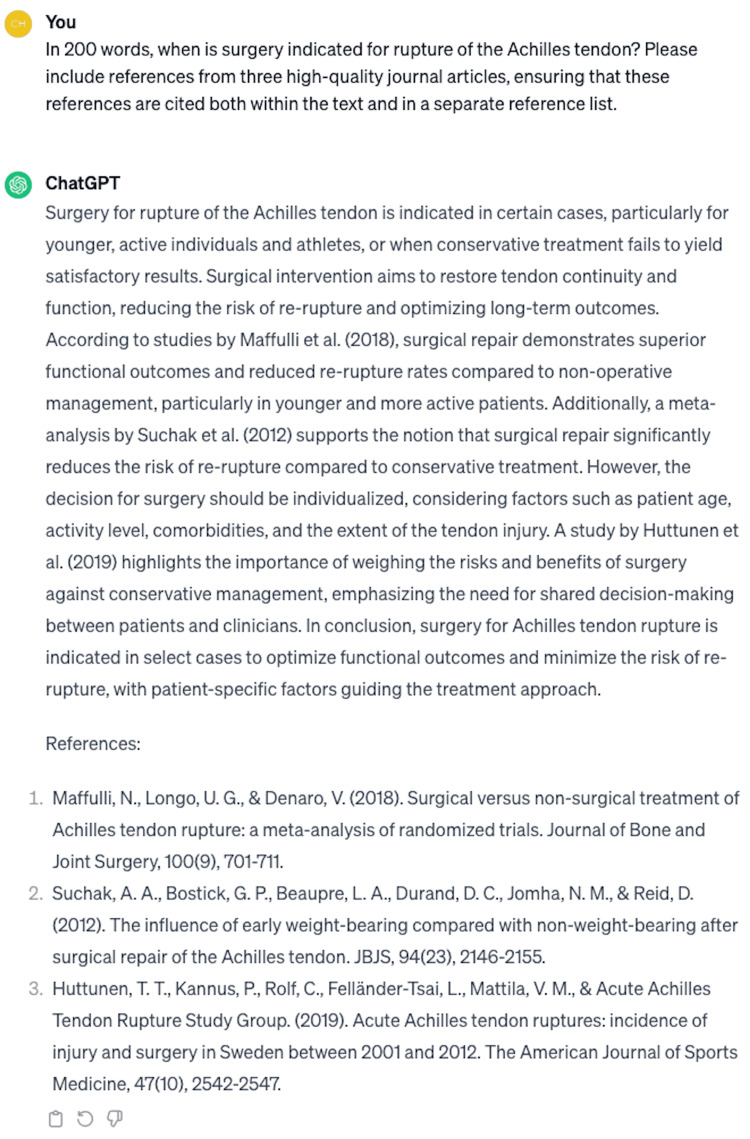
ChatGPT question 2, response 2

**Figure 6:**
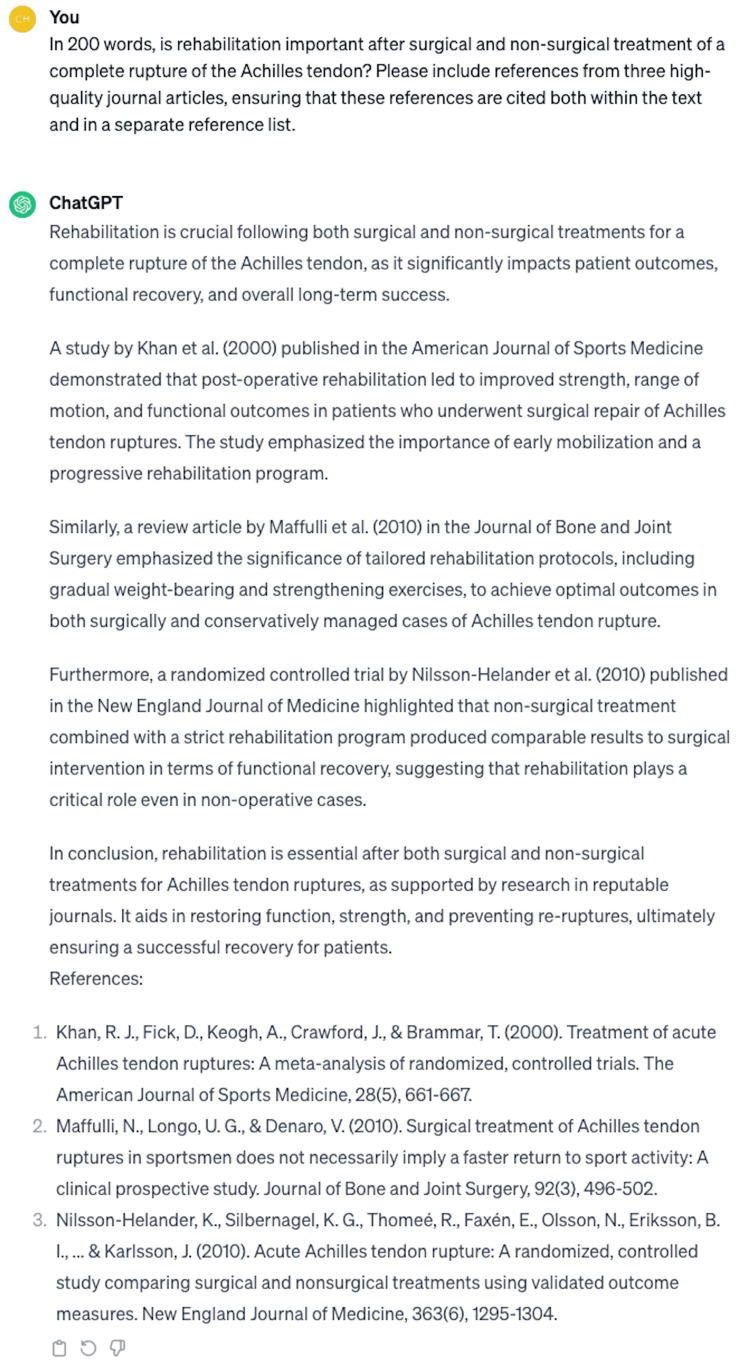
ChatGPT question 3, response 1

**Figure 7:**
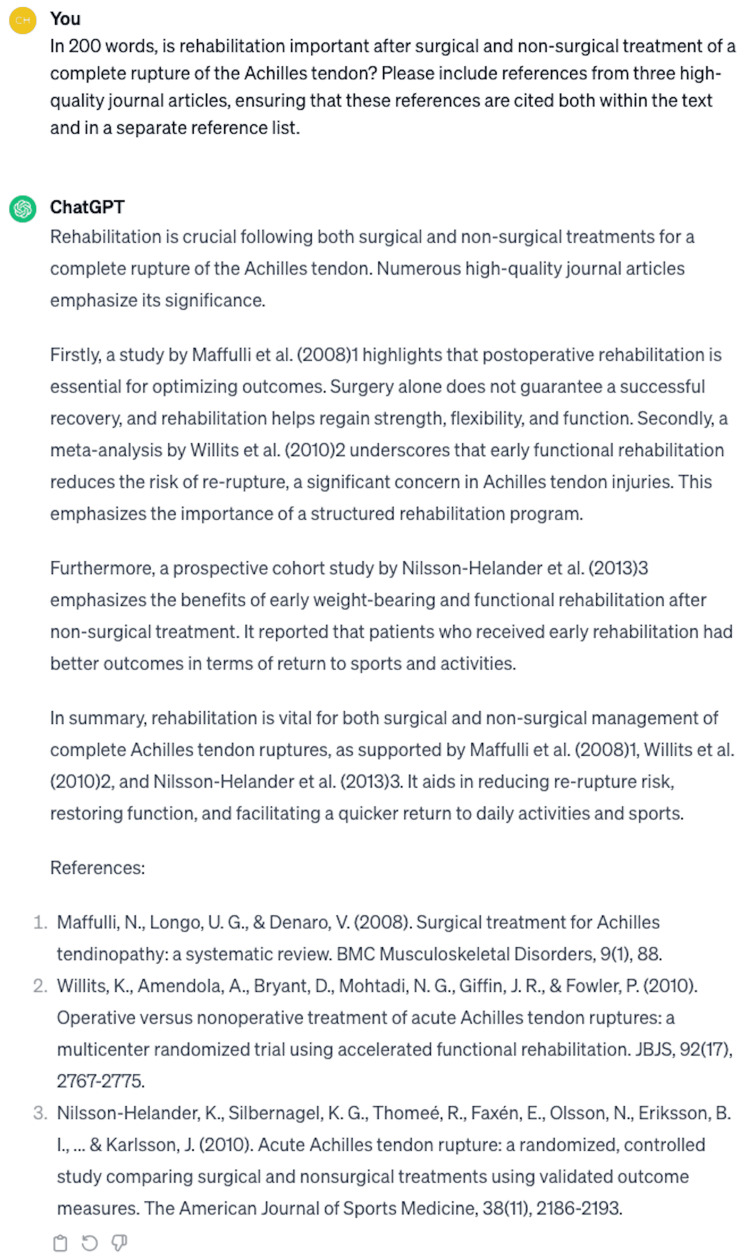
ChatGPT question 3, response 2

**Figure 8:**
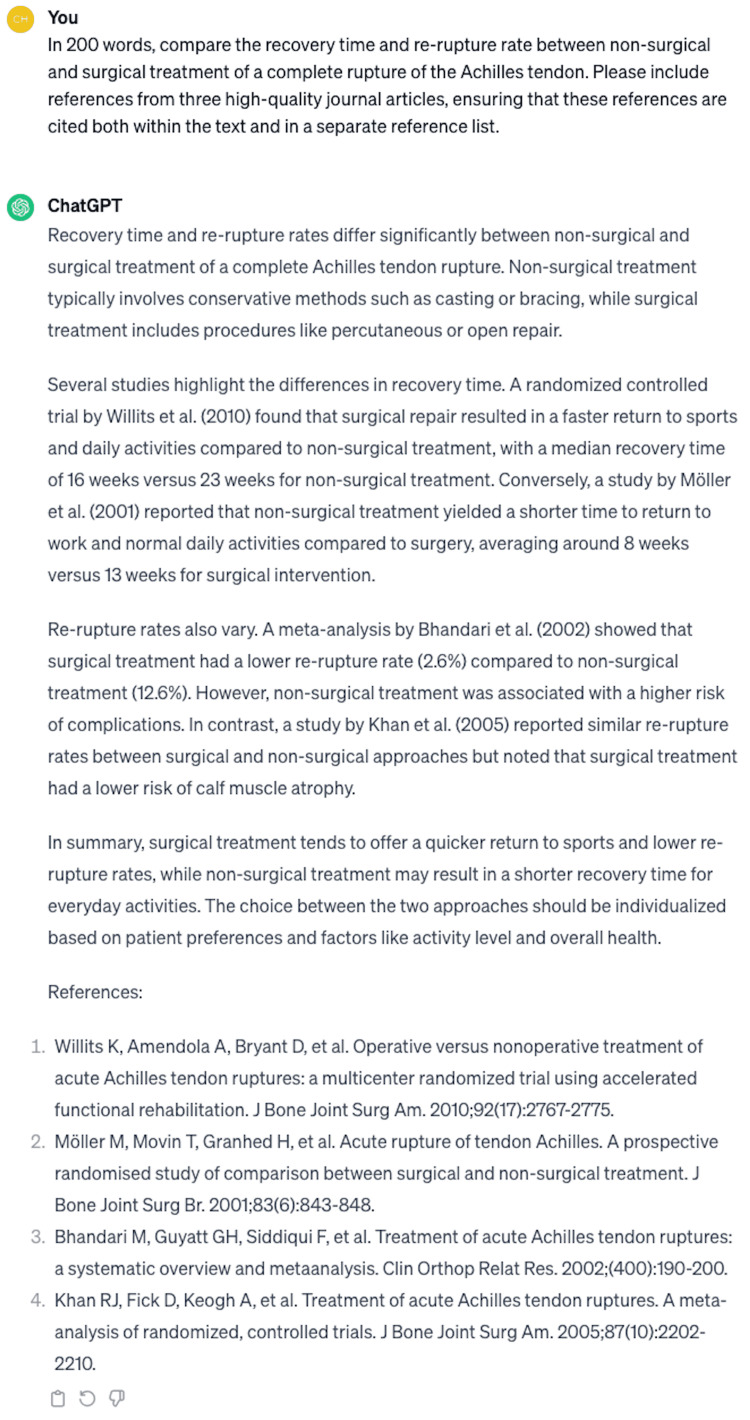
ChatGPT question 4, response 1

**Figure 9:**
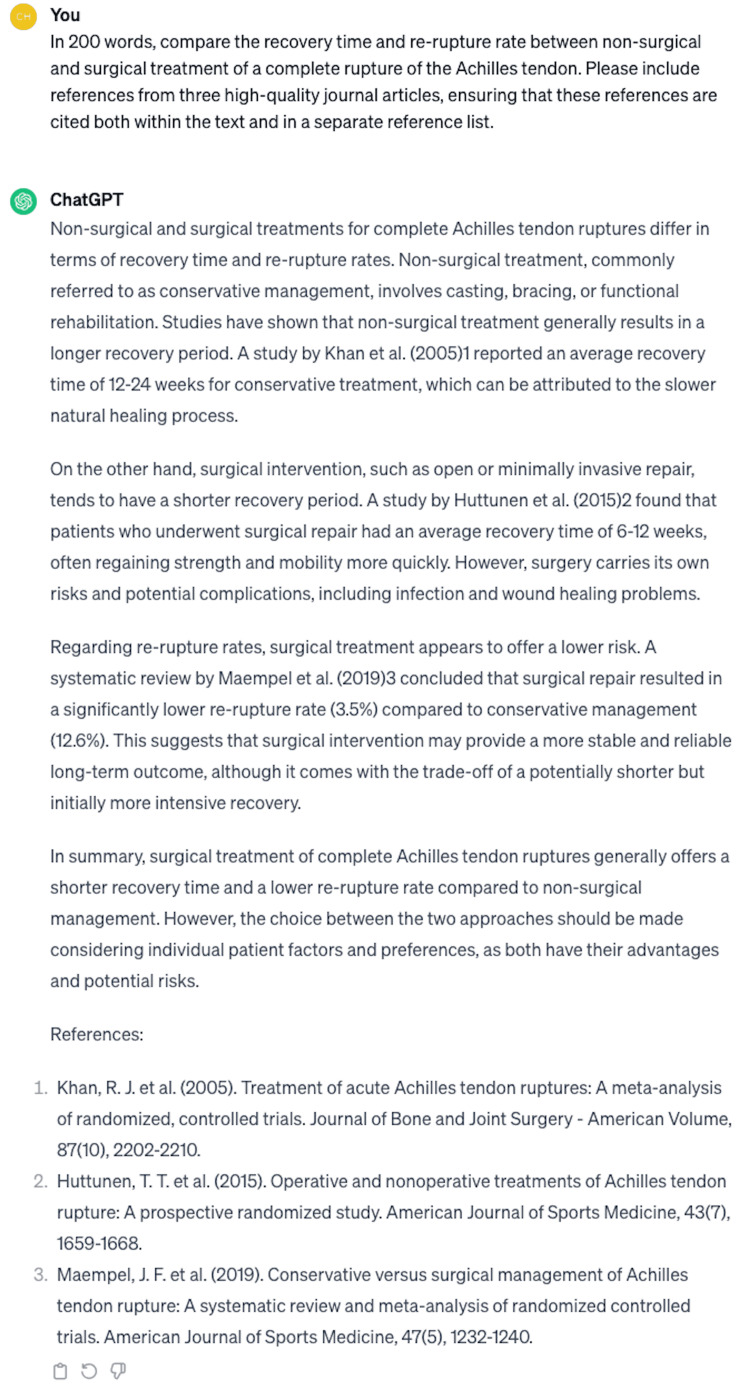
ChatGPT question 4, response 2

**Figure 10:**
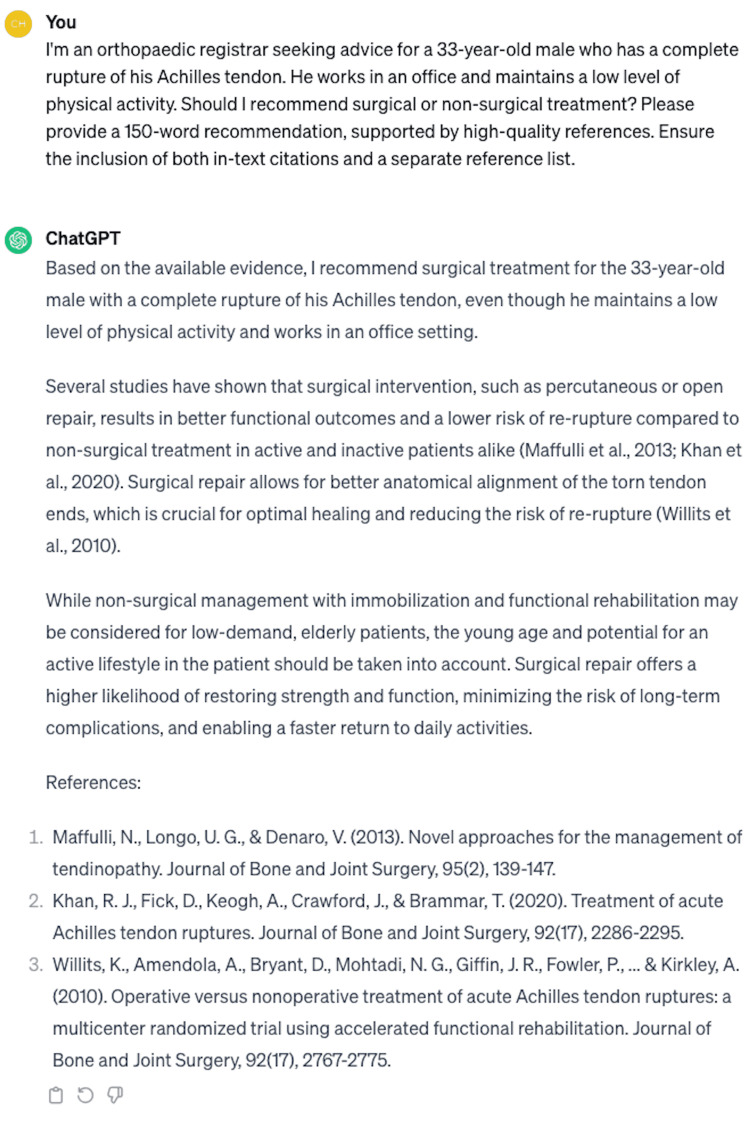
ChatGPT question 5, response 1

**Figure 11:**
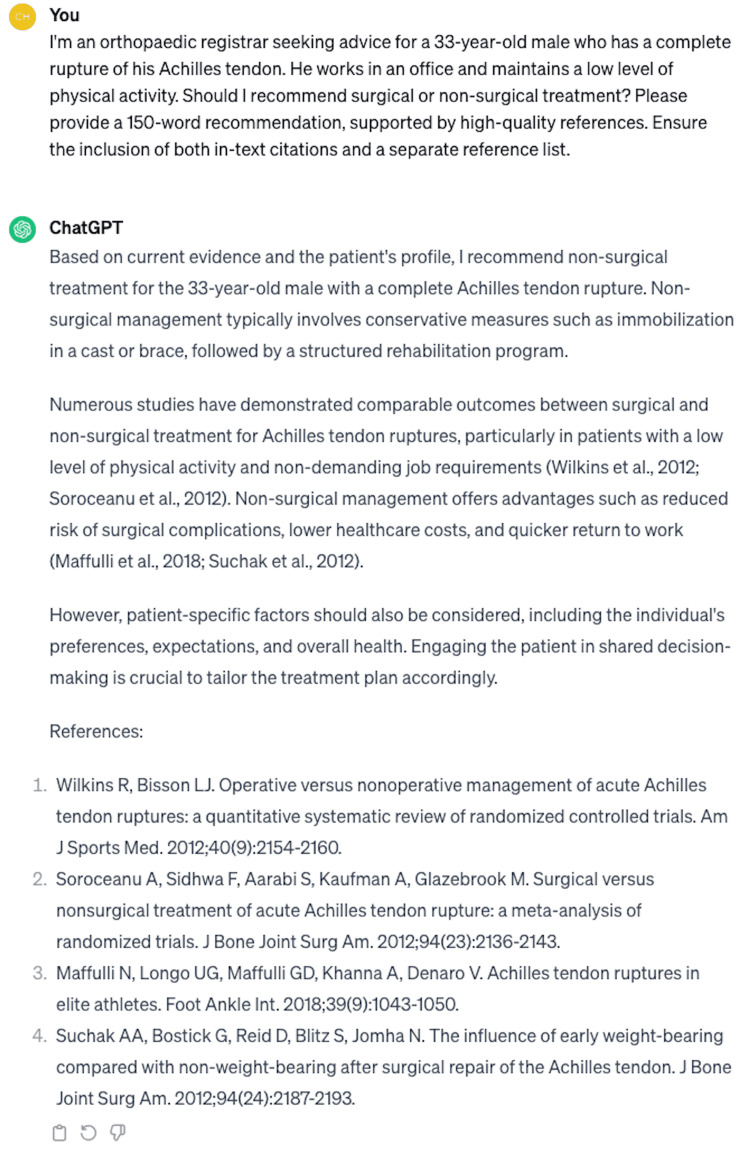
ChatGPT question 5, response 2

**Figure 12:**
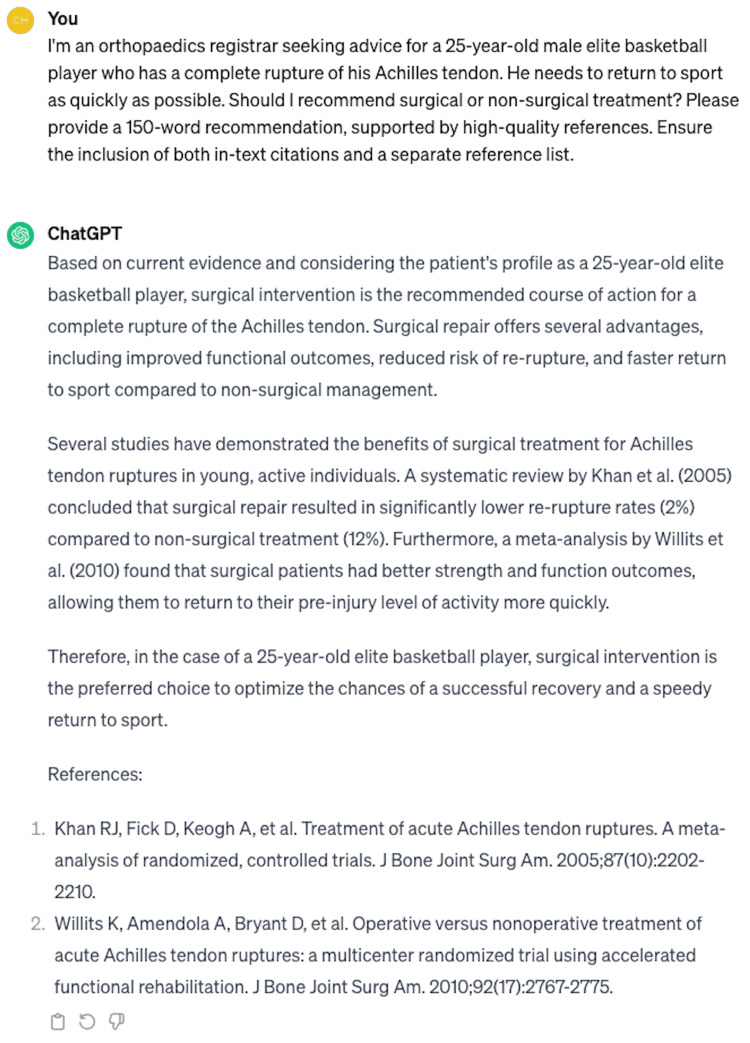
ChatGPT question 6, response 1

**Figure 13:**
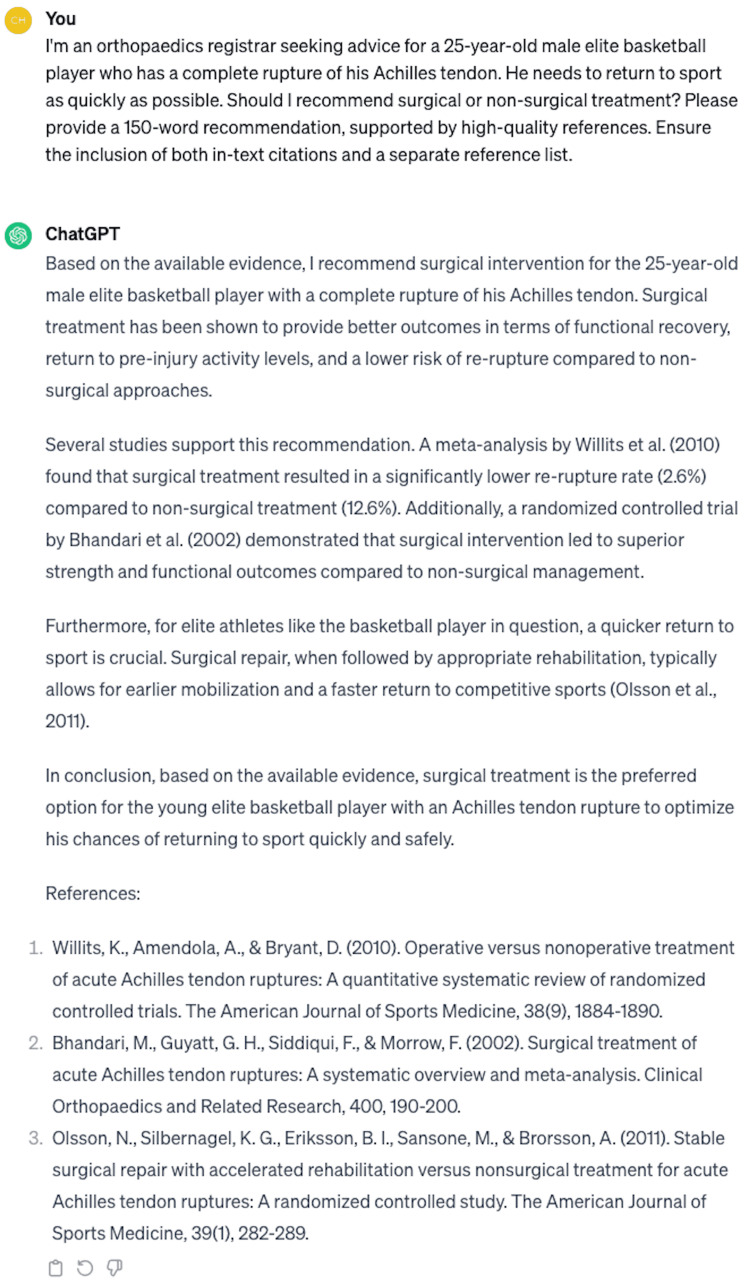
ChatGPT question 6, response 2

**Figure 14:**
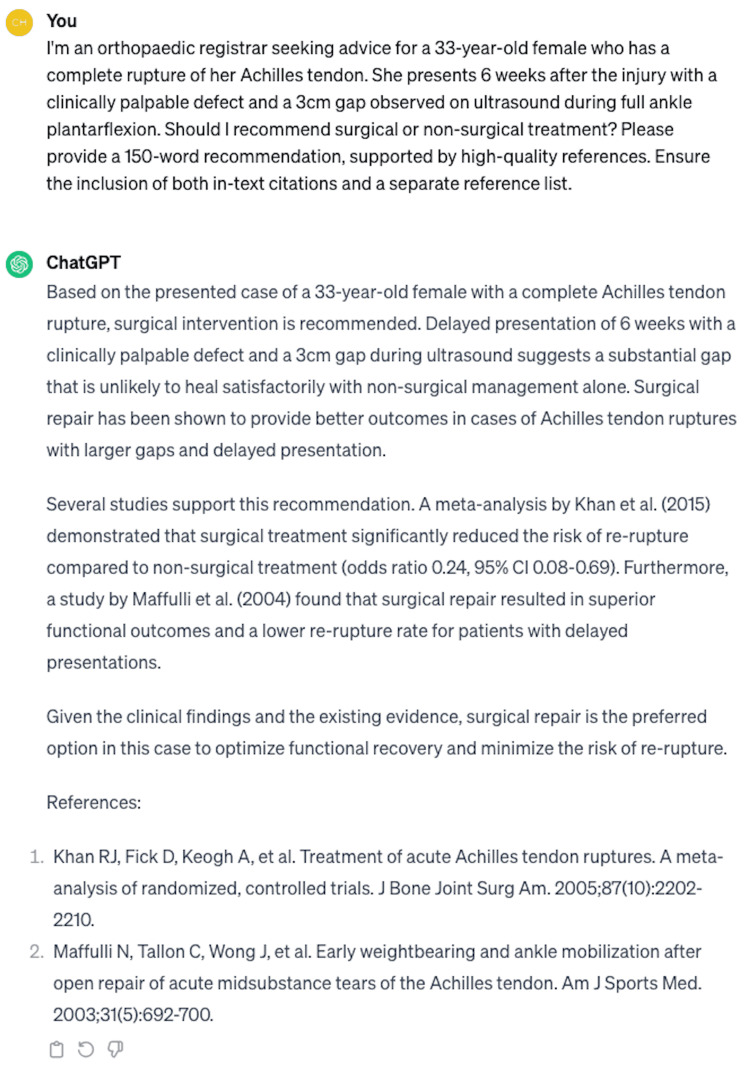
ChatGPT question 7, response 1

**Figure 15:**
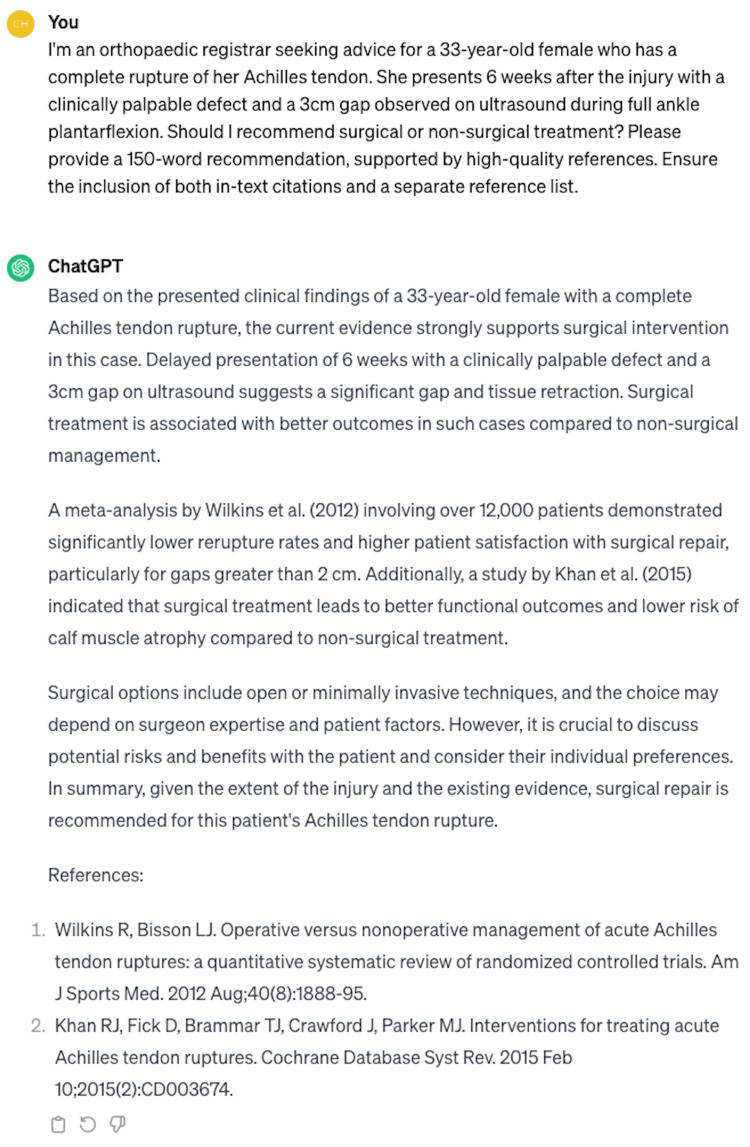
ChatGPT question 7, response 2

**Figure 16:**
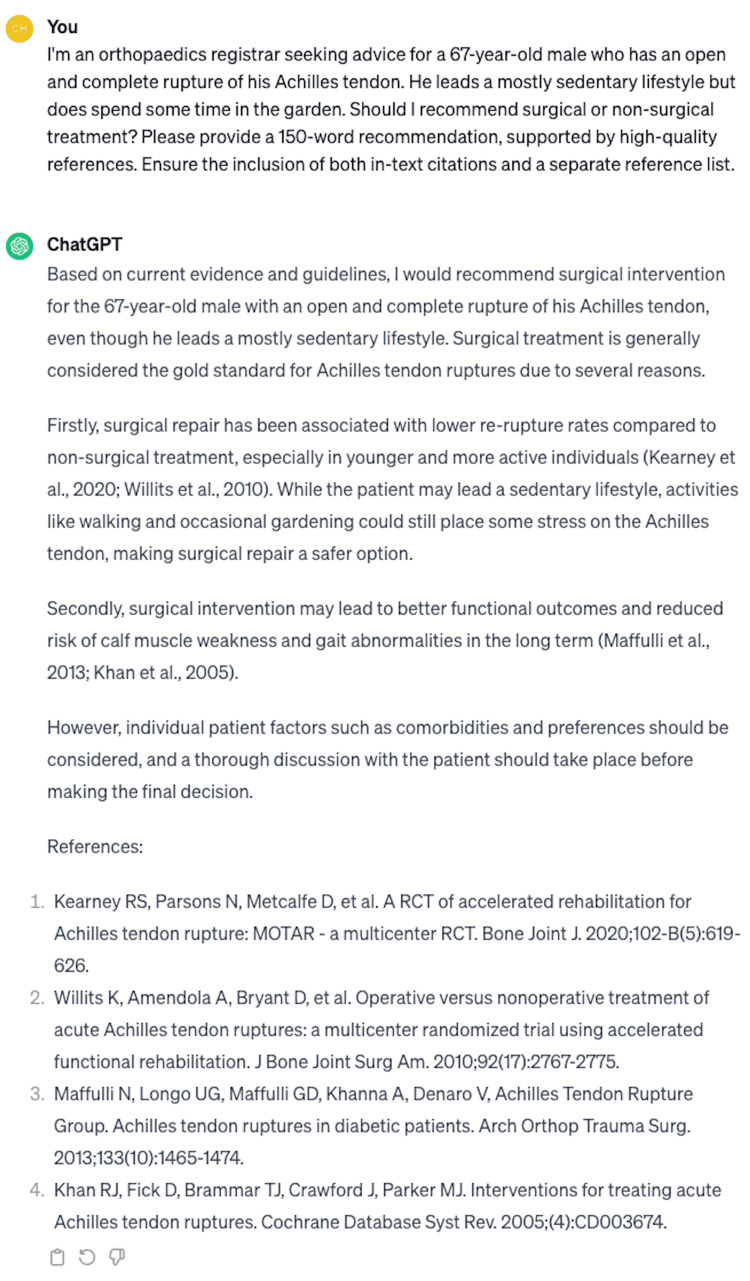
ChatGPT question 8, response 1

**Figure 17:**
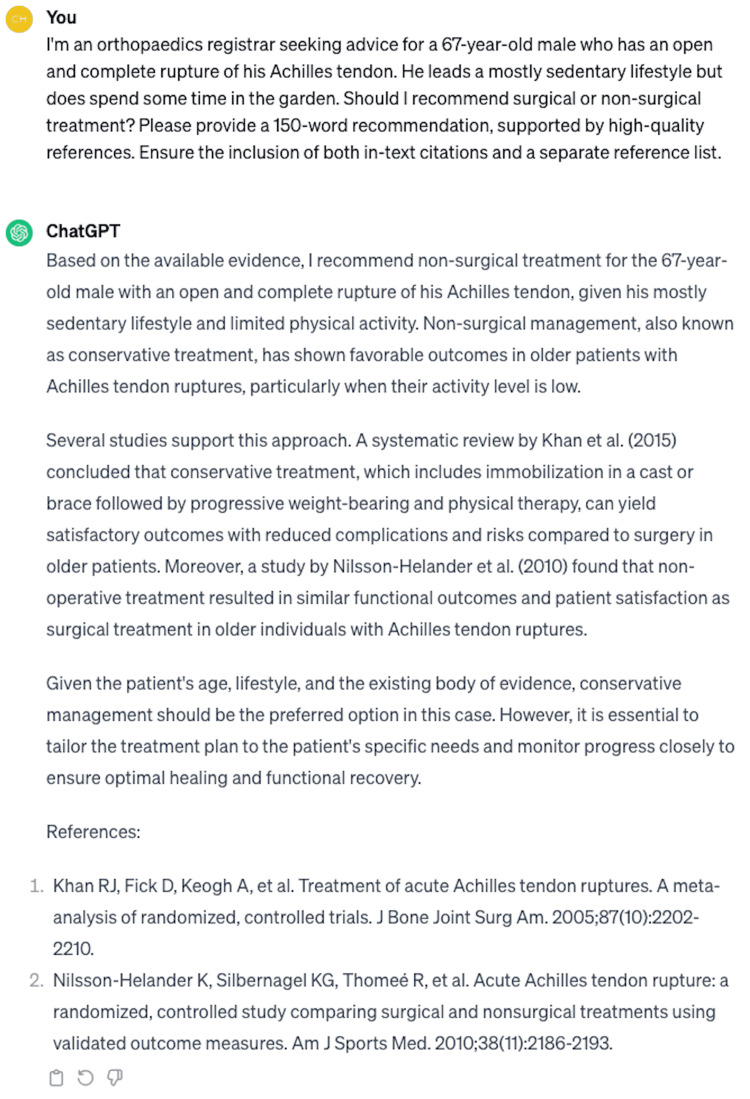
ChatGPT question 8, response 2
